# Health literacy and its related factors as predictors for the breastfeeding self-efficacy in a western province in Iran

**DOI:** 10.1186/s12889-023-15522-0

**Published:** 2023-03-30

**Authors:** Shahin Salarvand, Sepideh Ghazvineh, Fatemeh Mousivand, Hasan Ahmadi Gharaei, Saeid Bitaraf

**Affiliations:** 1 Hepatitis Research Center, Faculty of Nursing and Midwifery, Lorestan University of Medical Sciences, Khorramabad, Iran; 2 Student Research Committee, Lorestan University of Medical Sciences, Khorramabad, Iran; 3 Social Development and Health Promotion Research Center, Gonabad University of Medical Sciences, Gonabad, Iran; 4 Epidemiology Clinical Sciences Research Institute, Ahvaz Jundishapur University of Medical Sciences, Ahvaz, Iran

**Keywords:** Self-efficacy breastfeeding, Health literacy, Mothers

## Abstract

**Background:**

One of the effective factors on BF (Breastfeeding) continuation is Breastfeeding self-efficacy (BFSE). This study was conducted to determine the relationship between Health Literacy (HL) and BFSE in lactating mothers referring to primary health care centers.

**Methods:**

This cross-sectional descriptive study was carried out on lactating mothers referring to primary health care centers in 2022. Multi-stage cluster sampling was done with 160 samples.

The data were collected using demographic questionnaire, Persian shortened form of the BSES is a self-reported instrument for measuring a mother's Breastfeeding self-efficacy and Health Literacy for Iranian Adults (HELIA). Data were analyzed using ANOVA, independent t-test, correlation test and liner regression by SPSS version 16, with a significance level of 5%.

**Results:**

There was a significant positive correlation between the HL score and its four domains( Reading, Behaviour and decision making, Accessing, and Understanding) except for the appraisal domain with BFSE score. The variables of use of formula, HL, duration of breastfeeding, and education were considered predictors of BFSE.

**Conclusion:**

In general, the results indicate a possible relationship between BFSE and mothers' HL. Therefore, improving mother's HL can have a positive effect on promoting infants’ nutrition.

**Supplementary Information:**

The online version contains supplementary material available at 10.1186/s12889-023-15522-0.

## Background

Breastfeeding (BF), as a public health priority, is a key concept all over the world [1]. BF not only improves health in infancy but is also effective in ensuring human health in different stages of life and old age [2]. It reduces the possibility of anemia and vitamin deficiency in infants, and the risk of ovarian and breast cancer and diabetes in mothers [3, 4]. Lack of breastfeeding increases the risk of diseases such as respiratory infections, allergies, digestive problems, malnutrition, diabetes, and obesity [4].

As stronger links emerge between breastfeeding and lifelong disease prevention, breastfeeding promotion is increasingly becoming a priority for health agencies and among health care professionals [5]. In recent years, International World Health Organization(WHO) has stated that exclusive breastfeeding in the first 6 months of life brings the best physical and mental results for the baby [6]. The positive effects of breast milk increase with the increase in duration and its exclusivity [7]. One of WHO's goals in the field of infant nutrition achieves to increase the frequency of exclusive breastfeeding in the first 6 months of life to 50% in 2025 [8]. Therefore, early discontinuation and reduction of breastfeeding makes many harmful effects on child, maternal and public health and result in increased costs for the health care system [1].

Despite many efforts, breastfeeding is still far from optimal status in all worldwide. In diverse regions of the world, exclusive breastfeeding is low and many mothers prematurely discontinue [8]. Studies have shown that breastfeeding rates over time decrease to lower levels than in early practice [9–12]. Low rates and early cessation of breastfeeding have significant adverse health outcomes [9].

Given BF is a public health priority, descriptive studies to explain this phenomenon in different societies have addressed to identify risk factors and factors related to BFSE [4]. In general, the level of exclusive breastfeeding in countries and even different regions of a country is very variable. Also, racial and ethnic differences in infant feeding practices are significant, these differences have not been widely investigated regarding BF, maternal knowledge, and self-efficacy in infant feeding among low-income mothers [13]. More research is needed to find better ways to address BF disparities related to race and ethnicity, maternal knowledge, and infant feeding self-efficacy [13].

Culture and ethnicity are among the factors that affect health. Family, social and cultural influences are vitally important in shaping attitudes and beliefs and affect how people interact with the health system [14]. BFSE is an adjustable and effective variable on the initiation and continuation of breastfeeding [15]. Therefore, one of the ways to improve BF status is to adjust breastfeeding self-efficacy by identifying related factors [16]. BFSE is one of the constructs of social cognitive theory. In other words, it is the mother's belief and confidence in her ability to breastfeed her baby exclusively and successfully [17]. It is a valuable factor that predicts BF behavior and shows the mother's self-confidence and her ability to breastfeed. So that the higher the level of BFSE in mothers, the longer the duration of exclusive BF will be. This feature is a modifiable factor that can improve the amount and quality of breastfeeding [18]. Dennis believes that this increase in breastfeeding self-efficacy in mothers has a significant relationship with the increase in the duration of exclusive breastfeeding [19]. Moafi, et al., 's study showed that BFSE was the most effective factor in predicting exclusive BF in the third trimester after giving birth and it was able to increase the chance of exclusive BF [14]. However, a study by Hasanpoor et al. showed most mothers had low BFSE [20], and because of the low self-efficacy of BF, they do not continue BF as long as necessary [19]. The importance of BFSE is so great that many studies have been conducted on the factors affecting it and the effect of various interventions on it. In addition, two studies identified BFSE as a significant predictor of BF duration at two and six months postpartum [21, 22]. Likewise, breastfeeding self-efficacy is a prominent variable in breastfeeding duration, as it predicts; a. Does the mother decide to breastfeed? b. how hard she tries, c. Will she continue her efforts until mastery is achieved? d. Will she have self-defeating or self-enhancing thought patterns? and e. How she will react emotionally to breastfeeding challenges [21]. On the one hand, BFSE is influenced by factors such as anxiety level, mother's education level, understanding of the BF process, and knowledge and attitude towards BF [4]. And on the other hand, mothers with low BFSE are prone to prematurely discontinuation of BF [23] which they need to be supported [24]. It seems that another factor related to BFSE may be maternal health literacy(HL).

HL is an important factor in determining people's ability to understand, interpret and act on health information [25]. It is a dynamic concept [26]. which is recognized as a critical and modifiable factor in improving health outcomes and reducing health inequality [27]. The level of HL in Iran, based on the last national survey conducted in 2016, was estimated at 44%; That is, almost one out of every two Iranians had limited HL [28].

The key role of health literacy in benefiting from health care services and improving its outcomes has been well proven. HL is considered a basic foundation for the health and the life of modern citizenship. HL is a vital combination of social capital and should be considered as a policy not only in the health sector but in all sectors [29]. Low or inadequate HL is associated with multiple poor health outcomes [16]. Given women's health and knowledge have a direct effect on their family members and children, both before and during, and after childbirth; Mothers are the main focus of HL programs in the community [30]. According to the definition, maternal HL means social and cognitive skills that determine the mother's ability to obtain, understand and use the information to improve her and her children's health [31]. Today, BF promotion is highlighted as one of the most challenging aspects of HL (how to encourage people to use information in making health decisions) [32].

A review of the literature shows conflicting results about the relationship between HL and BFSE. As the results of Graus et al.'s study showed that 38.8% of mothers had insufficient HL, but they did not find a significant relationship between the level of HL and the duration of BF [13]. On contrary, the results of the study by Vila-Candel et al. showed that insufficient HL was one of the factors related to early discontinuation of BF, and it was recommended that more research is needed in this field [16]. Therefore, considering the importance of maternal HL and BF, defining predictors and risk factors that can make a significant contribution to improving mother and infant health is necessary [33]. Factors that are associated with BFSE and can predict it.

Since BFSE is one of the factors affecting the continuation of BF, both descriptive and interventional studies in this field continue and they are still looking for a solution to improve and increase this factor [34]. In addition, there is a paucity of extant studies investigating the relationship between HL and BFSE, as an important factor in BF behaviors. The need to address this issue and some factors affecting it can help health policymakers as an effective indicator. This study was conducted with aim of determining the relationship between HL and BFSE in lactating mothers referring to primary health care centers.

## Methods

This cross-sectional study was conducted in lactating mothers referring to primary health care centers in Khorramabad, Iran, from 18th December 2021 to 16th March 2022. The inclusion criteria were: Mothers with infants aged 0 to 2 years did not have any physical or mental illness or disorder that prevented BF and were willing to cooperate in completing the self-report questionnaires. Exclusion criteria included the incomplete response to the questionnaire. Multi-stage cluster sampling was done. There are 42 primary health care centers in Khorramabad city and approximately the number of lactating mothers is 15000. First, ten health centers among 42 primary health care centers from different locations were randomly selected. Then, 16 mothers were randomly selected from the list of lactating mothers in each center and asked them to complete questionnaires. The standard deviation (σ) of breastfeeding self-efficacy in the Charoghchian Khorasani et al., study was 11.7 [34]. The probability of type 1 error and the maximum acceptable difference in the mean were considered as 0.05 and 2, respectively. Assuming a 20% attrition and according to the following formula, the sample size was equal to 160.

$$n= \frac{{\left(\mathrm{z}1-\frac{\mathrm{a}}{2}\right)}^{2}* {\upsigma }^{2}}{{\mathrm{d}}^{2}}$$

The present study applied three questionnaires: a. Individual characteristics questionnaire, b. The Persian short form of the Breastfeeding Self-Efficacy Scale (BSES) [35], c. Health Literacy for Iranian Adults(HELIA) questionnaire [36]. Internal consistencies of mentioned instruments in the original study, Persian psychometrics study and current study were demonstrated in Tables S1 &  S2 (Appendix).

The breastfeeding self-efficacy scale is a 33-item questionnaire designed in 1999 by Dennis and his colleagues [37]. Dennis created its short form with 14 items in 2002 [38]. In 2014, Araban and his colleagues assessed the psychometric properties of this questionnaire in the Persian language and Iranian culture. This Persian version of BSES has 13 items [35]. A five-point Likert scale was used for scoring this instrument, ranging from "strongly agree" (five points) to "strongly disagree" (one point) [39]. The total score indicates higher levels of breastfeeding self-efficacy, with the least and highest possible scores of 13 and 65, respectively. The results of the psychometrics evaluation indicate BSES is an excellent instrument for measuring BFSE [38]. In the present study, we considered two years of BF duration. In the survey by Nanishi et al., a cutoff point equal to 50 was determined for this scale. it was divided into two groups of 50 and above(excellent level), and below 50(need to intervention) [40].

Health Literacy for Iranian Adults(HELIA) is an instrument to measure HL in Iranian people. This instrument was developed by Montazeri et al. in 2014 for Iranian population [41]. This questionnaire has a uniform structure and includes various aspects of HL, with multiple-choice items and is easy to use for the general population. This instrument consists of 33 items in 5 domains containing Reading (4 items), Appraising (4 items), Behaviour and decision making (12 items), Accessing (6 items), and Understanding (7 items). Each question is answered by a five points Likert-scale [1–5] [42]. The range of the HL scores is from 33 to 165. Scores between 33 to 66 are very poor HL, 67 to 99 are poor HL, 100 to 132 are Suitable HL, and 133 to 165 are very excellent. The questionnaire designers believe that one of the most important advantages of this tool is its usability for the general public. It means the tool does not belong to a specific population stratum and can be used for different demographic characteristics groups. It can measure the level of HL with acceptable accuracy [42].

Data are demonstrated in percentage, means (standard deviation). The normality assumption was checked. Linear regression, correlation test, ANOVA and independent t-test were used to analyze data. *P*-values under 0.05 were considered statistically significant. All analyses were done using SPSS version 16.

## Results

In the present study, the total scores of BFSE and HL and its domains in lactating mothers referring to primary health care centers were demonstrated in Table 1. Three participants(mothers) involved in the study were below 20 years old contains; two were 19, and one was 18 years old. In addition, 50% of mothers had Suitable health literacy and 25% of them had poor health literacy, and in general, the health literacy score of mothers was in the lowest range of the excellent level (133.36 ± 16.32). The mean score of BFSE was equal to 51.97 ± 8.54 (min–max = 25.00–65.00), and with a cut-off of 50 [40]. There were 62 mothers (38.8%) in the low BFSE group and 98 mothers (61.30%) in the high BFSE group (Table 1).

**Table 1:** Breastfeeding self-efficacy scores and health literacy and its dimensions in under-study mothers (*N* = 160)

	*Variable*	*Mean* ± *SD/ frequency(%N)*
*Breast feeding self-efficacy*	Total score of Breast feeding self-efficacy(13–65)	51.97 ± 8.54
*Breast feeding self-efficacy* *** level***	Excellent(≥50)	98 (61.3)
	Need to intervention(<50)	62 (38.8)
*Health literacy and its dimensions*	Reading domain(4–20)	16.59 ± 2.69
	Evaluating domain(4–20)	15.80 ± 2.60
	Behaviour and decision making domain(12–60)	45.96 ± 7.58
	Accessing domain(6–30)	23.81 ± 4.25
	Understanding domain(7–35)	31.18 ± 3.90
	Total score of Health literacy(33–165)	133.36 ± 16.32
*Health literacy level*	very poor (33–66)	5(%3.1)
	Poor (67–99)	40(%25)
	Suitable(100–132)	80(%50)
	Excellent(133–165)	35(%21.9)

In terms of demographic characteristics, the results of independent t-tests and ANOVA showed that mothers with older age, higher education, Employee, Private house and had health insurance coverage (Tamin ejtemaei/ Khadamate darmani) and had a HL literacy score than other groups. Breastfeeding rate decreased over time and mothers who breastfed longer had significantly higher BFSE. This is despite the fact that there was no statistically significant difference in the level of HL among these groups (Table 2).

**Table 2:** Health literacy and Breast feeding scores according to demographic characteristics variables of under-study mothers

*Variable*	*Categories*	*Frequency*	*Health literacy score*		*Breast feeding self-efficacy*	
			Mean ± SD	*P*-Value	Mean ± SD	*P*-Value
***Age***	<20	3	126 ± 13.85	0.01^*^	54.66 ± 9.54	0.32^*^
	20–29	52	127.94 ± 16.84		50.67 ± 7.57	
	30–39	88	136.53 ± 15.55		88 ± 52.09	
	>40	17	134.88 ± 15.54		17 ± 54.88	
***Education***	High school	28	119.75 ± 17.99	<0.001^*^	51.32 ± 8.33	0.06^*^
	Diploma	61	132.30 ± 13.75		53.93 ± 8.33	
	Bachelor's degree and higher	71	139.66 ± 14.24		50.54 ± 8.61	
***Occupational status***	Employee	41	142.29 ± 15.07	<0.001^**^	50.46 ± 9.12	0.19^**^
	Housekeeper	119	130.29 ± 15.65		52.49 ± 8.31	
***Marital status***	Married	156	133.54 ± 16.46	0.41^**^	52.09 ± 8.58	0.26^**^
	Divorced/Widowed	4	126.75 ± 7.84		47.25 ± 6.07	
***Housing status***	Private house	82	135.87 ± 15.70	0.04^**^	52.21 ± 9.01	0.71^**^
	Tenant	78	130.74 ± 16.65		51.71 ± 8.07	
***Type of insurance***	Tamin ejtemaei	72	135.61 ± 15.40	<0.001^*^	51.20 ± 8.98	0.39^*^
	Khadamate darmani	13	145.31 ± 17.22		56.30 ± 7.44	
	Roostaei	11	114.73 ± 17.32		52.90 ± 8.78	
	other	39	132.08 ± 14.31		51.64 ± 8.56	
	None	25	133.33 ± 16.32		52.04 ± 7.36	
***Gravida***	Once	52	131.81 ± 16.30	0.46^*^	50.40 ± 8.88	0.05^*^
	Twice	59	135.42 ± 17.26		51.35 ± 8.58	
	Three times and more	49	132.55 ± 15.22		54.38 ± 7.74	
***Para***	Once	62	131.27 ± 16.47	0.35^*^	49.70 ± 8.56	0.009^*^
	Twice	63	135.48 ± 16.59		52.44 ± 8.70	
	Three times and more	35	133.29 ± 15.54		55.14 ± 7.20	
***Duration of breastfeeding***	6 months>	54	133.28 ± 16.88	0.79^*^	49.22 ± 8.39	0.001^*^
	6–12 months	30	131 ± 15.52		50.13 ± 9.18	
	12–18 months	29	133.55 ± 14.25		53.37 ± 7.72	
	18–24 months	47	134.87 ± 17.64		55.44 ± 7.56	
***Formula feeding***	Yes	101	132.75 ± 16.60	0.53^**^	55.45 ± 6.70	<0.001^**^
	No	59	134.42 ± 15.93		46.01 ± 8.09	
***Health literacy***	Very low	5			45.80 ± 8.07	0.02^*^
	Low	40			49.40 ± 8.67	
	Moderate/Suitable	80			52.56 ± 8.43	
	Excellent	35			54.45 ± 7.86	

^*^ ANOVA—** independent t test

The variables of age, education, occupational status, Housing status, and type of insurance that had a significance level of less than 0.2 were entered into the final multivariable regression model and the stepwise method was used for analysis. The multivariable regression results showed that among these above-mentioned variables entered in the model (age, education, occupation, Housing status, and type of insurance), only the education variable was considered as a predictor of HL, which predict for 18% of the total variance of the HL variable. As, by increasing one unit of education from high school to diploma as well as higher education, the HL score increases by 0.43 units (Table 3).

**Table 3:** Linear Regression results for Factors predicting Health literacy in mothers

*Predictor variables*	*Unstandardized B*	*SE*	*Standardized B*	*T test*	*P-Value*
***Education***	9.47	1.58	0.43	5.98	*P* *<0*.001
	R = 0.43	R^2^ = 0.185	Adj R^2^ = 0.18		

In general, the breastfeeding self-efficacy score in the present study was reported to be higher than the mean (51.97 ± 8.54) (Table 1). The results of the correlation test showed that there is a significant positive correlation between the HL score and all its domains except the Appraisal domain with BFSE (*P<*0.05) (Table 4).

**Table 4:** Correlation between health literacy and its domains with breastfeeding self-efficacy

Variable		Reading domain	Appraisal domain	Behavior and decision making domain	Accessing domain	Understanding domain	Total score of Health literacy
**Breast feeding self-efficacy**	r	0.24	0.12	0.31	0.24	0.16	0.31
	*P*-Value	0.002	0.12	<0.001	0.002	0.04	<0.001

The results of independent t-tests and ANOVA showed that mothers with three or more pregnancies (*P* = 0.05), three or more successful pregnancies (*P* = 0.009), longer breastfeeding duration (*P* = 0.001), not using formula feeding (*P<*0.001), and had a higher HL score (*P* = 0.05), and had a higher BFSE score than other groups (Table 2).

The variables of education, occupation, number of gravidae, number of paras, duration of breastfeeding, use of formula feeding, and HL that had a significance level of less than 0.2 were entered into the final multivariate regression model and the forward method was used for analysis.

The results of multivariable regression showed that among the variables interred in the model (education, occupation, number of gravida, number of paras, duration of breastfeeding, use of formula feeding, and HL), the variables of using formula feeding, health literacy, duration of BF and education as the predictors of breastfeeding self-efficacy were addressed, which predict 38% of the total variable variance of BFSE. So that mothers who used formula for their babies, compared to those who did not use formula, their HL score decreased by 0.48 units, and with an increase of one unit in the HL score of mothers, the BFSE increased by 0.31, with an increase of one unit in the duration of BF increased the BFSE score by 0.18, and with the increase of one unit of education, the BFSE score decreased by 0.16 (Table 5).

**Table 5:** Linear Regression results for factors predicting breastfeeding self-efficacy in under-study mothers

*Predictor variables*	*Unstandardized B*	*SE*	*Standardized B*	*T test*	*P-Value*
***Formula feeding***	-8.49	1.13	-0.48	-7.48	<0.001
***Health literacy***	3.51	0.77	0.31	4.54	<0.001
***Duration of breastfeeding***	1.26	0.44	0.18	2.84	0.005
***Education***	-1.86	0.80	-0.16	-2.32	0.02
	R = 0.63	R^2^ = 0.40	Adj R^2^ = 0.38		

## Discussion

This study was conducted to determine the relationship between HL and BFSE in lactating mothers referring to primary health care centers. In the present study, the BFSE score was higher than the Mean (51.97 ± 8.54). Moafi et al. also reported the BFSE score in hospitalized women during 24 h after delivery of 57.28 ± 7.65 in Qazvin [2]. which was higher than the Mean too. in contrast, the results of Hasanpour et al.'s study showed low BFSE score in most mothers in Ahvaz [23]. Although there is cultural and religious homogeneity in Iran, it seems the quality and lasting of breastfeeding vary in different areas and depend on ethnicity and sub-cultures. In the present study, 50% of mothers had a suitable HL and 25% of them had a poor HL, and in general, the Mean score of mothers' HL was in the lowest range of the excellent level (133.36 ± 16.32). In two studies by Hosseini et al., the level of health literacy of mothers was reported as suitable level [43, 44]. Mirjalili et al. reported HL level of most mothers(%71.8) was suitable and excellent [45]. The results of the studies of Qudsizadeh and Peyman showed that the level of HL of most lactating mothers was poor [46, 47]. It seems the reason of high health literacy in the present study is the educated mothers and the extent of internet resources and social networking.

In terms of demographic characteristics, the results of the present study showed that employees, private house, and the kind of health insurance coverage had a higher HL score than other groups. The results of the Vila-candle’s study confirmed that female employees had a higher level of health literacy [16]. This could be because employed mothers have higher education and social interaction, and more health literacy than non- employed mothers/housekeepers, and they can obtain scientific and reliable materials. No relevant study was found about owning a private home and health insurance coverage and its relationship with the level of health literacy.

There was a significant relationship between the mother's age and the level of HL, so older mothers had a higher HL score (*P* = 0.01). The results of Peyman's study confirm that there is a significant relationship between age and the level of health literacy [47]. In contrast, Mirjalili’s study showed no significant relationship was found between HL level and Mothers’ age [45]. This may be because older mothers have more experience.

The present study showed that the variable of education was considered a predictor of HL So that with the increase in education, the score of HL increased. And mothers who had higher education had a higher HL score (*P<*0.001).

Also, in the present study, among the variables included in the model, only the education variable was considered as a predictor of HL. Other studies also confirm that a significant relationship was found between a mother's education level and HL [48].

The present study showed breastfeeding rate decreased over time, and mothers with higher BFSE had significantly breastfeeding longer. This is despite the fact that there was no statistically significant difference in the level of HL among these two groups.

In general, the BFSE score in the present study was reported to be excellent level (51.97 ± 8.54). Other studies confirmed and reported the mean BSES-SF score were excellent level [46, 49]. It means the mothers evaluated themselves in a high ability in breast feeding process.

In the present study, no significant relationship was found between the mother's age and BFSE. This finding may be due to the vast access to medical information in the current era via mass media and the internet, and more social support and empowerment of mothers in breastfeeding as its outcomes. So that Mothers in every age can be informed. Although, several studies showed that increasing maternal age was associated with the higher BFSE [50] or a longer duration of BF and increased chances of BF [44, 48]. In contrary, the results of Khabazkhoob et al.’s study showed that as the mother's age increases, exclusive BF decreases significantly [51].

The results of the present study showed that mothers with three or more pregnancies, three or more successful pregnancies, longer BF duration, no use of formula feeding, and a higher health literacy score, in comparing with other groups, had a higher BFSE score. The present study showed that the variables of not using of formula feeding, HL, duration of BF, and education were considered predictors of BFSE. So, mothers who used formula feeding for their babies had a lower BFSE score than those who did not use formula feeding. Ngo, et al.’s study reported the self-efficacy of breastfeeding was significantly higher in mothers who breastfed their babies than in mothers who fed them with formula [50]. The results of the correlation test showed that there is a significant positive correlation between the HL score and all its domains except the appraisal domain with BFSE. The BFSE score increased with the increase in the HL score of mothers. Other studies have provided conflicting results. Some studies have confirmed this finding and some have rejected it. Several studies found a direct and significant relationship between the HL score and increased duration of BF and exclusive BF [52]. In contrast, Graus et al.’s study reported that there is no significant relationship between the level of HL and the duration of BF [13]. Kaufman et al. reported that women with a higher HL level were more likely to initiate BF and continue exclusive BF for the first 2 months after delivery [52]. It seems that mothers who have a higher level of HL are more aware of the importance of BF, have better access to resources, and are better able to overcome BF difficulties and to have higher BFSE.

In the present study, it was shown that as the duration of BF of mothers increased, their BFSE scores increased. No study related to this finding was found, but it is obvious that mothers who breastfed longer were more successful during BF. It is evident that the higher BFSE, the more the breastfeeding duration. Dennis, et al., believe that the higher BFSE in mothers causes a longer BF period [37, 53].

In present study, As mothers’ education level increased, the BFSE score decreased. The results of other studies are inconsistent with this finding. Khabazkhoob et al.’s study showed that exclusive BF has an inverse relationship with a mother's education and exclusive BF is significantly more in mothers with lower education level [51]. The study by Charoghchian Khorasani et al. reported no significant relationship was observed between the mean score of BFSE and educational level, and occupational status [54]. Iranian mothers with a high level of education may not have enough time to breastfeed their babies, and another reason is they don’t like to make burnout in their bodies through this breastfeeding process.

In contrast, the results of the study by Lechosa-Muñiz et al. indicated that children have been born to mothers with university education levels were 53% more likely to be breastfed than their counterparts who were born to mothers with primary education [48].

The limitations of this study include; that BFSE and HL were self-reported by mothers. In addition, the sample population is limited, and this study was conducted on women referring to healthcare centers, which makes it difficult to generalize the results. the research was conducted in a single city in the western area of Iran with its own culture. Another limitation of the present study was its cross-sectional nature, which does not facilitate the process of interpreting the time sequence of relationships between variables.

## Conclusion

In general, these results indicate the possible relationship between BFSE and women's HL; Self-efficacy can be affected by HL. Inadequate knowledge about a specific health issue, due to poor HL, may affect people's self-efficacy regarding their ability to adhere to a care plan for themselves and their child. Therefore, improving maternal HL can have a positive effect on promoting children's BF nutrition. It is recommended to use HL strategies to improve the mothers’ HL in the community. The most important strength of this study was to investigate the relationship between breastfeeding self-efficacy and maternal health literacy in Iran for the first time. It is worth mentioning that this meaningful relationship can be used as a useful strategy in planning educational interventions.

## Supplementary Information

**Additional file 1: Table S1.** Internal consistencies of BFSE instrument in the original study, Persian psychometrics study and current study. **Table S2.** Internal consistencies of HELIA instrument in the original study, Persian psychometrics study and current study.

## Data Availability

All the data generated during this study are included in this article.

## Abbreviations

BSES: Breastfeeding Self-Efficacy Scale
HELIA: Health Literacy for Iranian Adults
HL: Health literacy
BF: Breastfeeding
