# Supplementary material for: Health literacy and its related factors as predictors for the breastfeeding self-efficacy in a western province in Iran
Source: BMC Public Health. 2023 Mar 30;23:593. doi: 10.1186/s12889-023-15522-0 (PMC10061825; doi:10.1186/s12889-023-15522-0)
Supplement: Supplementary file 1 — Additional file 1: Table S1. Internal consistencies of BFSE instrument in the original study, Persian psychometrics study and current study. Table S2. Internal consistencies of HELIA instrument in the original study, Persian psychometrics study and current study. [file 12889_2023_15522_MOESM1_ESM.docx]

**Appendix:**

**Table S1:** Internal consistencies of BFSE instrument in the original study, Persian psychometrics study and current study

| *Breastfeeding self-efficacy scales* | *Alpha Cronbach* |
| --- | --- |
| *short form in Persian in current study* | 0.902 |
| *validated short form in Persian* | 0.91 |
| *original short form* | 0.93 |

**Table S2:** Internal consistencies of HELIA instrument in the original study, Persian psychometrics study and current study

|  | *Alpha Cronbach* | | | | | |
| --- | --- | --- | --- | --- | --- | --- |
|  |  | *dimensions* | | | | |
| *Persian health literacy scale* | *total* | *reading* | *Evaluating* | *behavior* | *accessing* | *understanding* |
| *original HELIA* | *Not reported* | 0.72 | 0.79 | 0.87 | 0.78 | 0.89 |
| *HELIA in current study* | 0.93 | 0.87 | 0.87 | 0.88 | 0.74 | 0.85 |
